# Do Images of ‘Watching Eyes’ Induce Behaviour That Is More Pro-Social or More Normative? A Field Experiment on Littering

**DOI:** 10.1371/journal.pone.0082055

**Published:** 2013-12-05

**Authors:** Melissa Bateson, Luke Callow, Jessica R. Holmes, Maximilian L. Redmond Roche, Daniel Nettle

**Affiliations:** 1 Centre for Behaviour and Evolution, and Institute of Neuroscience, Newcastle University, Newcastle upon Tyne, United Kingdom; 2 School of Psychology, Newcastle University, Newcastle upon Tyne, United Kingdom; Utrecht University, Netherlands

## Abstract

Displaying images of eyes causes people to behave more pro-socially in a variety of contexts. However, it is unclear whether eyes work by making people universally more pro-social, or by making them more likely to conform to local norms. If the latter, images of eyes could sometimes make people less pro-social if pro-social behaviour is not the local norm. To separate these hypotheses we conducted a field experiment in which we explored whether manipulating a local descriptive norm altered the eyes effect. We recorded litter dropping decisions on a university campus in a 2 x 2 design, comparing situations with and without litter already on the ground (a manipulation of the local descriptive norm) and with and without large signs displaying images of watching eyes. We additionally recorded the number of potential human observers in the vicinity at the time of each littering decision. We observed a norm effect: the presence of litter on the ground increased littering, replicating previous findings. We also found that images of watching eyes reduced littering, although contrary to previous findings this was only when there were larger numbers of people around. With regard to our central aim, we found no evidence that litter on the ground interacted non-additively with images of eyes to induce increased littering behaviour. Our data therefore support the hypothesis that images of eyes induce more pro-social behaviour, independent of local norms. This finding has positive implications for the application of eye images in combating anti-social behaviour.

## Introduction

There is growing evidence that engaging the psychology of surveillance using simple images of watching eyes induces people to behave more pro-socially. This effect, henceforth the ‘watching eyes effect’, was first demonstrated in controlled laboratory experiments employing economic games such as the Dictator and Public Goods Games [1-3]. A number of subsequent studies using similar methods have demonstrated that participants are more likely to transfer money to others in the presence of an image of eyes compared with a control image [1,4-7]. Positive watching eyes effects have also been reported on a number of different real-world decisions. In the presence of images of eyes people are more likely to pay for their drinks via an honesty box [8], donate to a charity bucket [9] and recycle appropriately [10]. They are also less likely to leave litter on cafeteria tables [11] or steal bicycles from a university campus [12]. These results raise the possibility that cheap interventions based on simple images of watching eyes could be used to tackle anti-social behaviour, and even crime, in a range of real-world situations. However, before such interventions are widely adopted, we need to understand the psychological mechanisms underlying the watching eyes effect, since this could influence the class of situations in which images of watching eyes are most likely to have positive impacts on decision making. Here we explore whether local behavioural norms influence the watching eyes effect.

The motivation for our study question comes from the fact that there are multiple interpretations for what drives the watching eyes effect. One simple possibility is that that the effect of watching eyes will always be to induce more pro-social behaviour. This can be linked to ‘reputation-based partner choice’ models of the evolution of cooperation in humans [13,14]. Under these models, people are pro-social, in the absence of immediate returns, as an investment in their social reputations. A good reputation in turn increases the likelihood of being favoured by others for inclusion in future mutually-beneficial interactions. Being observed increases the reputational consequences of an action, and hence, people are psychologically sensitive to whether they are observed or not, and will always increase their level of pro-sociality when observed over their level when not observed. Artificial watching eyes exploit this sensitivity.

A subtly different possibility invokes ‘norm psychology’ [15]. Norm psychology consists of sensitivity to locally-specific behavioural norms, coupled with a tendency to sanction departures from these norms. The presence of observers increases the perceived probability of being sanctioned for departing from local norms. Thus, being watched (or artificial images that exploit the feeling of being watched) should make people more normative. Norm psychologists distinguish two different types norm: an injunctive norm is an action that most people would approve, whereas a descriptive norm is what most people actually do [16]. Theoretically, both types of norm could be affected by cues of surveillance. The pro-sociality hypothesis and the norm psychology hypothesis often predict the same outcome. For example, when it is locally normative to behave pro-socially, then both hypotheses predict that watching eyes will increase pro-sociality. However, there are situations where the local norm is not to be pro-social, and in such situations, the norm psychology hypothesis would predict that watching eyes would *decrease* pro-social behaviour, whereas the pro-sociality hypothesis would predict them to *increase* it*.*

Some support for the norm psychology hypothesis comes from a recent meta-analysis of the watching eyes effect in the Dictator Game [4]. Whilst watching eyes make people more likely to give something, they do not increase the mean amount given, because the variance in donations is reduced under eyes. These data are compatible with the hypothesis that images of eyes make people more normative, giving an amount of money closer to the mid-point of possible donations, which represents some kind of perceived norm, as opposed to making them uni-directionally more generous. However, set against this, Powell, Roberts and Nettle [9] found that watching eyes strongly increased charitable donations in a context (charity collection buckets in a supermarket) where most people did not donate (there was no descriptive norm of donation), and there was no sanction for not donating (there was no injunctive norm to donate). They interpret their findings as lending more support to the pro-sociality than the norm-psychology hypothesis for the watching eyes effect. 

The strongest test between the pro-sociality and norm psychology accounts of the watching eyes effect would be to experimentally manipulate which behaviour is locally normative, and test for an interaction between local norms and the presence of watching eyes. Our aim in the current paper was to carry out such an experimental test by making use of an established methodology for experimentally manipulating a descriptive norm. We focussed on littering behaviour for a number of reasons. Littering is an extremely costly societal problem and there is considerable interest in cheap interventions that could reduce it [17]. Littering is additionally an easily quantified behaviour that can be observed and manipulated in real-world situations [16]. We have previously demonstrated that littering of tables is reduced by images of watching eyes in a self-clearing cafeteria with an established norm of litter clearing [11]. However, there is also substantial evidence that littering behaviour is strongly affected by local descriptive norms. Littering behaviour leaves a physical mark on the environment (litter) that acts as a cue to the local prevalence of littering behaviour. This feature makes it possible to manipulate cues of the local descriptive littering norm without the need for people to actually witness the behaviour itself. It has previously been demonstrated that people are more likely to drop litter if there is already litter present on the ground or if there are other cues of disorderly behaviour such as graffiti in the local environment, and moreover, these effects have been shown experimentally by manipulating the amount of litter present in the local environment [16,18,19]. 

**Figure 1:**
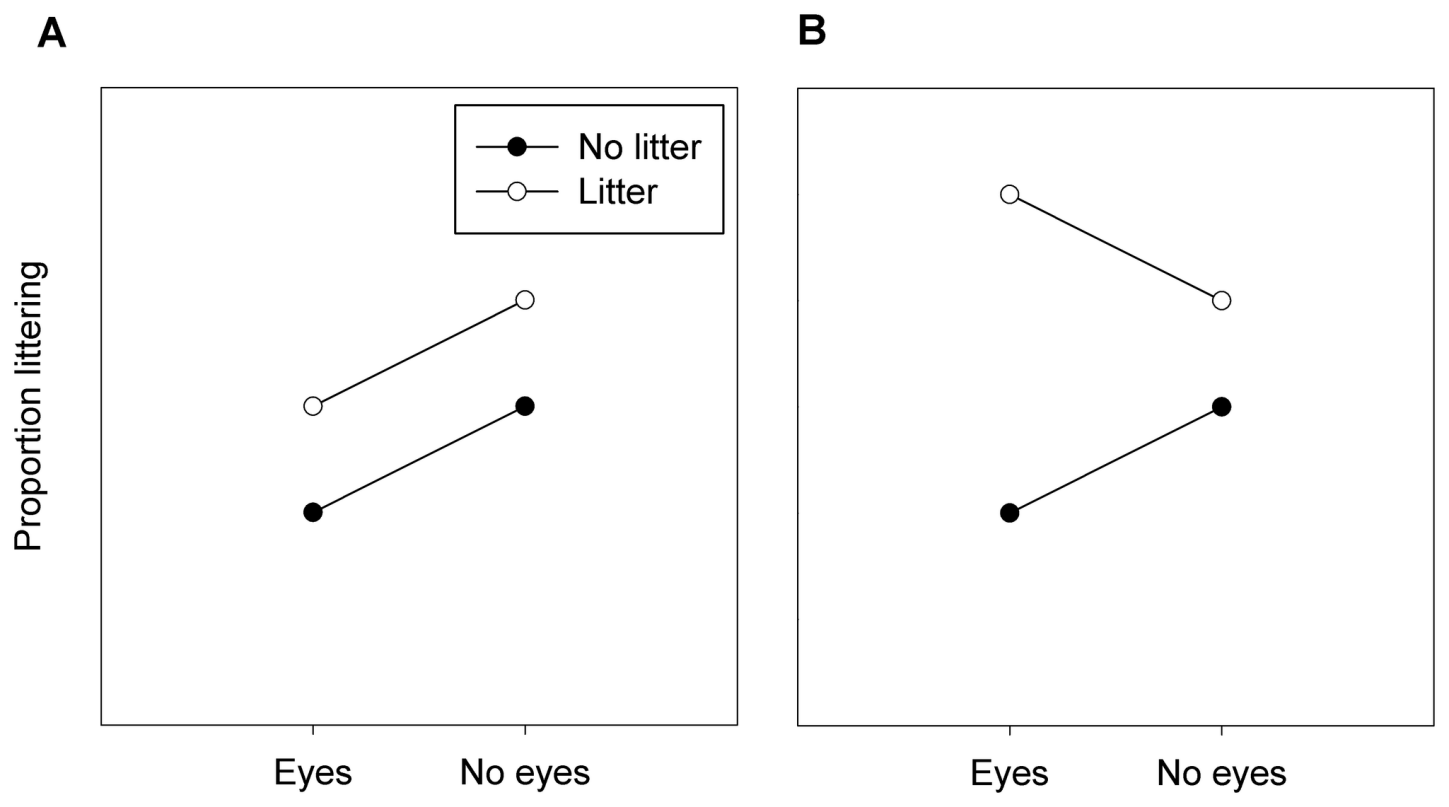
Theoretical predictions. Graphical representation of alternative predictions: (a) eyes and litter have additive effects on littering behaviour; (b) there is a non-additive interaction between eyes and litter, whereby eyes enhance the effects of litter such that the highest proportion of littering is seen when both eyes and litter are present. Alternative patterns of results are also possible, but for the purpose of this study it was these two hypotheses that we sought to separate.

The above results suggest that it should be possible for us to test the prediction that if watching eyes induce more normative behaviour, littering behaviour will *increase* in the presence of images of watching eyes when there is already litter present in the environment and hence littering is perceived as locally normative. To test this prediction, we conducted a field experiment with a 2 x 2 factorial design in which we quantified littering behaviour either in the presence or absence of images of eyes, and simultaneously, either in the presence or absence of litter already present on the ground. If images of eyes always induce more pro-social behaviour we predicted additive main effects of eyes and existing litter: littering behaviour should be independently increased in the absence of eyes or the presence of litter (Figure 1a). In contrast, if eyes induce more locally normative behaviour, we predicted a non-additive interaction between eyes and existing litter such that littering is most likely when both eyes and litter are present (Figure 1b). We additionally recorded the number of people present in the vicinity at the time of each littering decision, because previous studies have shown that the eyes effect can be modulated by the number of real people potentially observing a decision [9,11,20].

## Methods

### Ethics statement

Ethical approval for the study was obtained from Newcastle University’s Institute of Neuroscience Human Psychology Ethics Committee (application number 000401). Since no individuals were approached or identified during the study and the participants were simply observed in a public place, it was not considered necessary or appropriate to obtain informed consent or conduct debriefing. Our ethics committee waived the requirement for written informed consent.

### Study site and participants

The experiment took place at six bicycle racks on the campus of Newcastle University. We chose bicycle racks as the location for the experiment because three racks had durable signs featuring a large pair of staring eyes (60 x 90 cm) preinstalled on the walls above the racks, apparently looking down over the bicycles (see pictures in [12]). These signs had been installed approximately 18 months previously as part of an anti-bike theft campaign and additionally bore a verbal message irrelevant to the current study. The other three racks used in the experiment had no signs installed and acted as control locations without images of watching eyes. The control racks were chosen to be similar in size to the racks with eyes. All six bicycle racks were situated near to the entrance of major university buildings that were heavily used by staff and students during the period of the study. All six racks had a litter receptacle in the vicinity, which is important since probability of littering is positively related to the distance from a bin [21]. All locations also had good artificial lighting meaning that visibility of both the signs and other people was maintained after sunset. 

The participants in the experiment were all cyclists, and are likely to have been largely students and university staff. They comprised 439 males and 181 females and were all judged to be between the ages of 18 and 40. 

### Generation of littering opportunity

**Figure 2:**
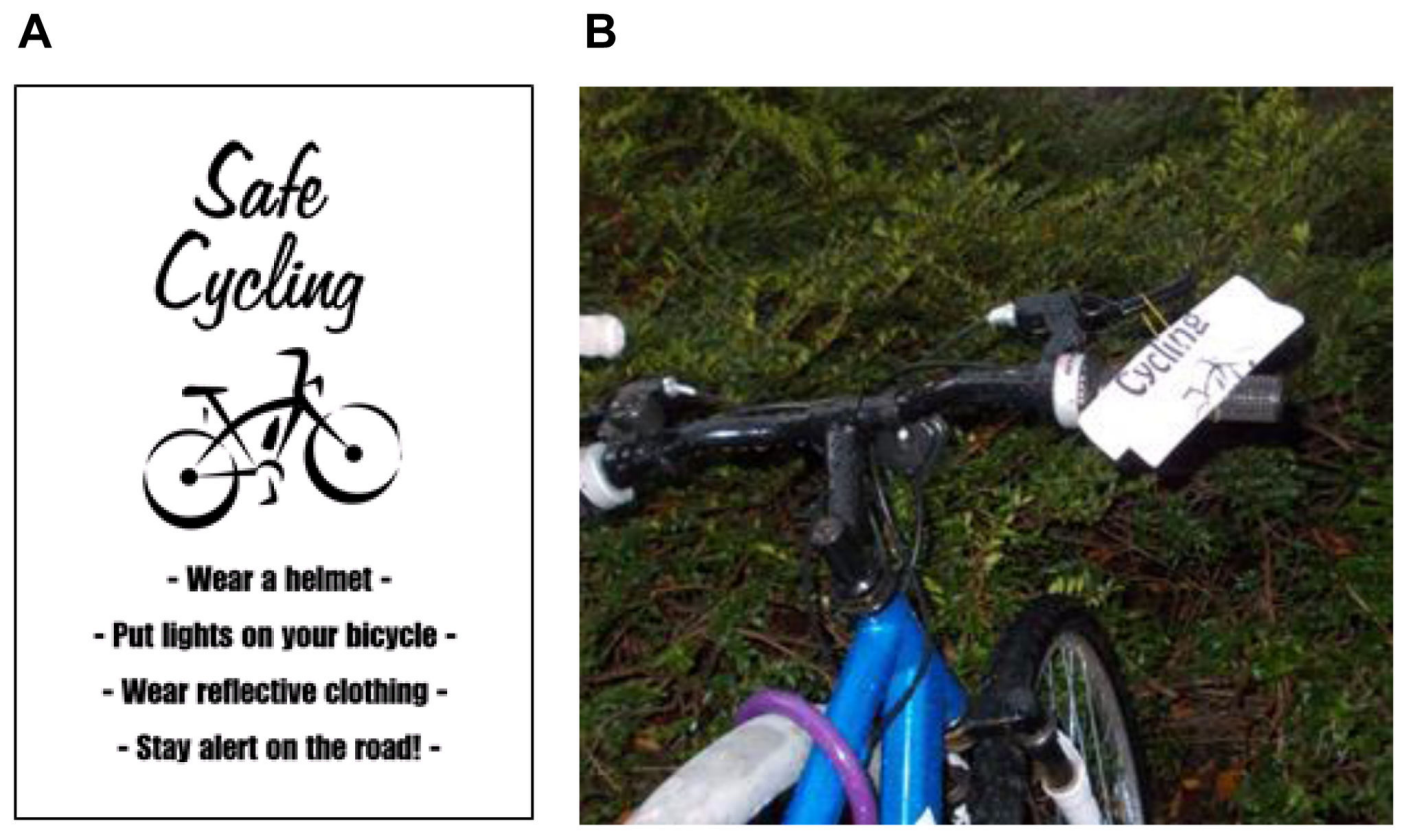
Leaflet and method of attachment. (a) The leaflet used, and (b) an example of how the leaflets were attached to the bicycle handlebars.

To create a standardised opportunity to litter, we attached a leaflet to each bicycle parked in the rack at the beginning of each observation period and to any new bicycle that arrived during the observation period. The leaflet was printed in black on white paper and contained suggestions for safe cycling (Figure 2a). It was designed to resemble a genuine flyer that was relevant to bicycle users, but contained no information that might cause the reader to want to keep it. The flyer was attached to the handlebars with an elastic band in such a manner that it would be difficult to move the bicycle without first removing it (Figure 2b). All leaflets still attached to bicycles at the end of each observation period were removed.

### Manipulation of littering norm

In order to change the perceived descriptive norm for littering we experimentally manipulated the amount of litter present on the ground during our observation periods. At each bicycle rack we had two conditions. In the ‘no litter’ condition we removed all existing litter from between and around the bicycle rack prior to the beginning of each observation period and also at intervals within the period. In the ‘litter’ condition we artificially increased the litter between and around the bicycle rack prior to the beginning of each observation period. The litter we used was comprised of screwed-up and regular leaflets, both the experimental leaflets (see above) and random advertising leaflets (e.g. takeaways, local businesses). It also contained sweet wrappers and empty drinks cans. The litter was scattered randomly around the area of the bike rack; the highest concentration of litter was nearest the centre of the bicycle rack and this decreased in concentration gradually up to approximately 2 m away from the rack in all directions. At the end of each observation period all experimental litter was cleared away.

### Data recording and analysis

A single observer (either LC, JRH, or MLRR) recorded the data from an inconspicuous position near to each bicycle rack (sometimes inside an adjacent building). Inter-observer reliability was established prior to the start of formal data collection and the three observers contributed equally to data collection across the experiment. The observers were not blind to the treatment combination in place. However, whilst they were aware of the published main effects of watching eyes and litter on littering behaviour, at the time of data collection we had not agreed the specific hypothesised interaction between these variables that is the focus of this paper. Hence, it is unlikely that the observers’ data were influenced by the main hypothesis under test (i.e. that eyes would increase littering when littering was cued to be normative).

Each person returning to collect one of the leafleted bicycles became a participant in the experiment and provided a single data point. Their behaviour towards the leaflet was categorised as follows: they either left without removing it, kept it on their person (e.g. put it in a pocket or bag), placed it in a nearby litter bin, placed it elsewhere in the vicinity (e.g. on an adjacent bicycle or window sill), or dropped it on the ground. Additionally, we recorded the apparent sex of the person, the approximate age of the person (categorised as either <18, 18-25, 26-40 or 40+) and the approximate number of other people within a radius of approximately 6m of the participant at the time of the littering decision (categorised as either 0, 1-5, 6-10, 11-15 or 16+). Data were collected from each of our six locations on a total of four different days, two days with litter absent and two days with litter present. The six locations were used sequentially in a repeating cycle with the litter condition alternating between cycles. The observation periods were each of 2 hours duration and took place between 1130 and 1730 on 24 days between 25/10/12 and 13/12/12.

For the purposes of the analysis each data point (littering decision) was assumed to be independent. Whilst it is possible that the same individual could have been observed more than once, this is relatively unlikely because the pool of potential participants was very large (Newcastle University has more than 20,000 students, and employs over 5,000 staff), and the bike racks were adjacent to large buildings on the main campus heavily used for teaching. Furthermore, data collection at each location was spread over four days and six hours of the day in order to capture different populations of people leaving the buildings at the end of classes. Hence we believe the assumption of independence is reasonable, but acknowledge the possibility of some non-independence as an unavoidable limitation of the study. 

Data were analysed in SPSS version 19. Our dependent variable was the decision to litter. Cases where the participant left without removing the leaflet were coded as missing values, since we could not be sure that the participant had noticed the leaflet and their decision could not be reliably classified as littering or not littering. Dropping the leaflet on the ground was coded as ‘littering’, keeping it on the person, placing it in a bin or elsewhere in the vicinity were all coded as ‘not littering’. Since our dependent variable was binary (littered/did not litter), we used generalised linear models with a binomial probability distribution and a logit link function in order to model the effects of our various predictor variables. An alpha value of 0.05 was assumed throughout.

## Results

The raw data from the study are available as Supporting Information (Data S1 and Data S2). We observed a total of 620 people returning to a bicycle to which we had attached a leaflet. The behaviour of these participants, and how we classified their littering decisions, are summarised in Table 1. 

**Table 1:** Summary of behavioural decisions and how these were classified.

**Behavioural decision**	**N**	**%**	**Classification**	**N**	**%**
Left without removing leaflet	18	2.9	Data discarded	-	-
Dropped leaflet on the ground	163	26.3	Littered	163	27.1
Put leaflet in nearby bin	39	6.3	Did not litter		
Put leaflet elsewhere in vicinity	74	11.9	Did not litter	439	72.9
Retained leaflet on person	326	52.6	Did not litter		
Total	620	100		602	100

**Figure 3:**
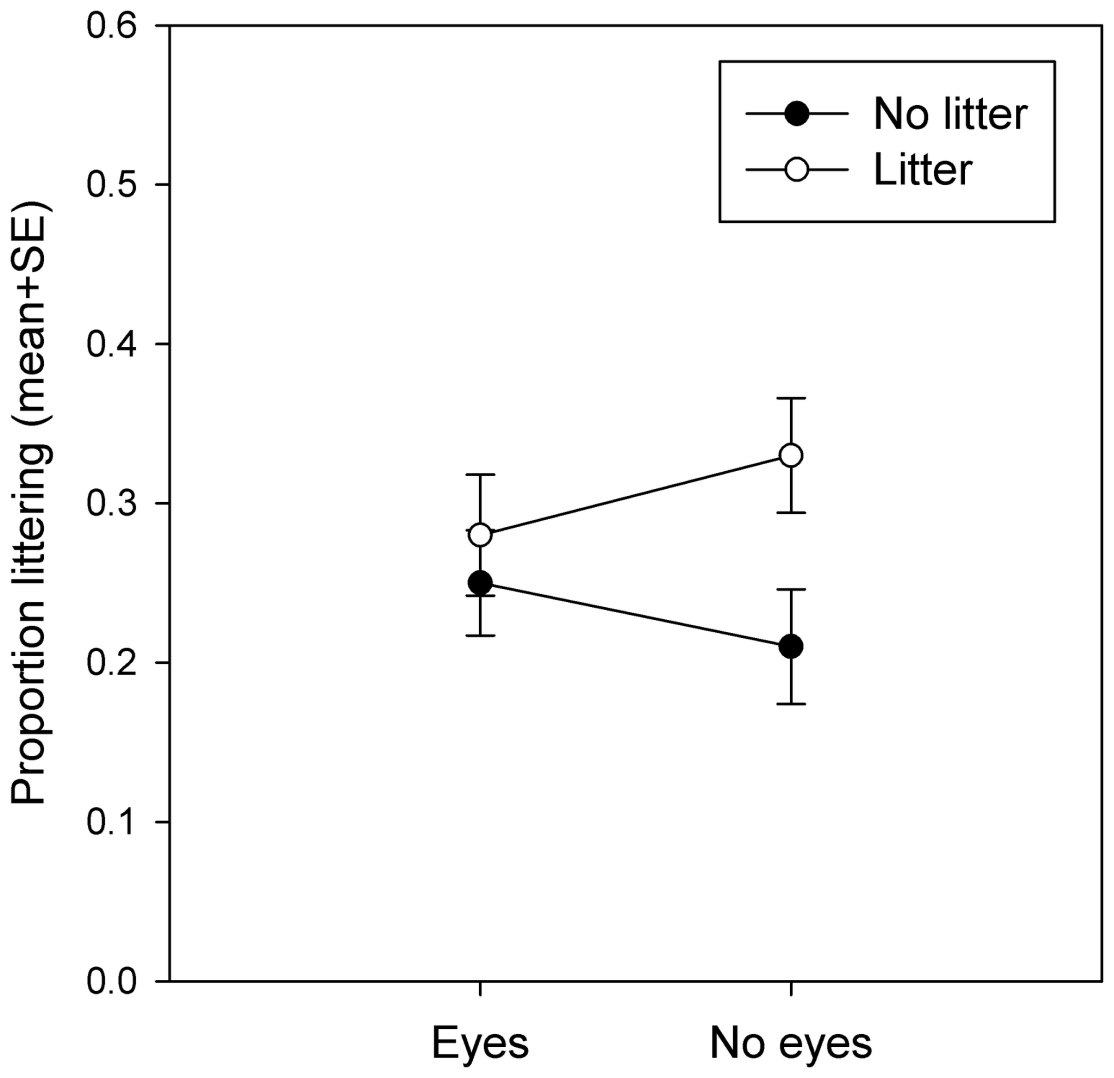
Proportion of participants littering in each treatment combination. Note that the highest level of littering is seen in the ‘no eyes’/’litter’ condition as predicted in Figure 1a by the hypothesis that effects of eyes and litter are additive. Graph shows estimated marginal means from the 2 x 2 generalised linear model+SE.

To test for main effects of images of eyes and existing litter on the ground we performed a generalised linear model with the decision to litter (littered/did not litter) as the dependent variable and the presence of eye posters (eyes/no eyes), the presence of litter on the ground (litter/no litter) and the ‘eyes x litter’ interaction as categorical predictors. There was a significant main effect of litter (Wald χ^2^ =4.214, df=1, p=0.040), with a greater proportion of people dropping litter when there was litter present on the ground than when litter was absent. However, littering behaviour was not significantly affected by eyes (Wald χ^2^=0.002, df=1, p=0.964), or the ‘eyes x litter’ interaction (Wald χ^2^=1.707, df=1, p=0.191; see Figure 3.). 

**Figure 4:**
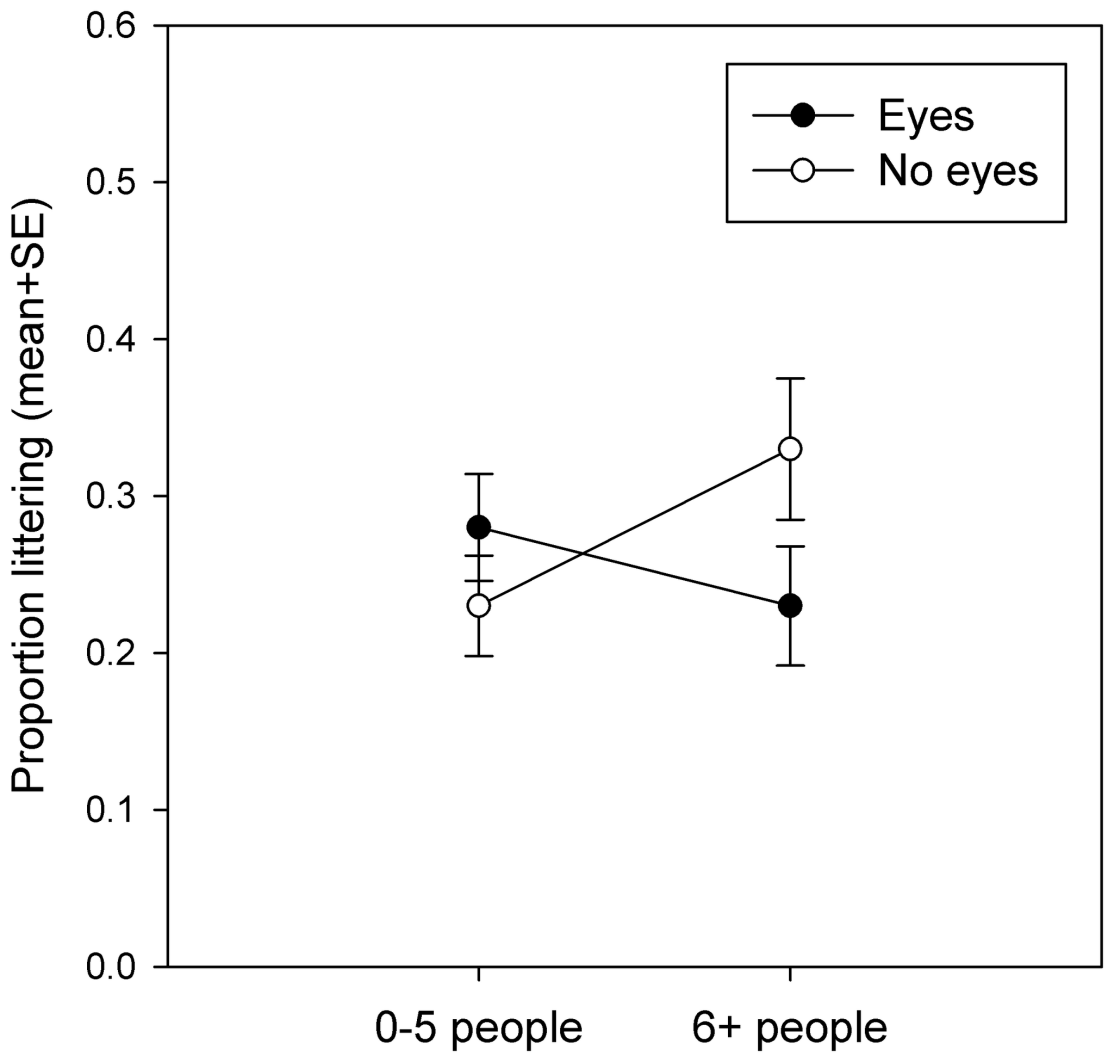
Interaction between watching eyes and number of people. The proportion of participants littering is significantly predicted by the interaction between the number of people in the vicinity and the presence of signs with images of watching eyes. The interaction appears to be driven by a near-significant (p=0.060) pairwise comparison between 0-5 and 6+ person groups in the ‘no eyes’ condition: littering increased when more people were present in the ‘no eyes’ condition only. Graph shows estimated marginal means from the second generalised linear model+SE.

Next, we explored whether an interaction with the number of people present could be obscuring a watching eyes effect in the current data set. Since the number of observations that fell into each of the five categories we recorded for number of potential observers was very unequal, we formed a new variable, ‘people’ by classifying decisions into two groups: those made when there were 0-5 people in the vicinity (n=373), and those made when there were 6 or more people in the vicinity (n=247). We repeated the generalised linear model described above with the ‘eyes x people’ interaction as an additional categorical predictor of littering behaviour. Since models must be hierarchical, it was also necessary to include the main effect of ‘people’. In this model, the interaction of ‘eyes x people’ was the only term that explained significant variation in the proportion of people littering, and the effect of ‘litter’ was now marginally non-significant, probably due to lack of power (Table 2), but the pattern was the same as that seen in Figure 3. (estimated marginal mean with litter present+SE=0.30+0.03; estimated marginal mean with litter absent+SE=0.23+0.03). Post-hoc pairwise comparisons indicated that the ‘eyes x people’ interaction was driven by a rise in the proportion of people littering when there were more people around in the ‘no eyes’ condition only (p=0.060; see Figure 4). As in the simple 2 x 2 model presented above, the ‘eyes x litter’ interaction was not significant and there was no indication of the predicted interaction between eyes and litter shown in Figure 1b; the interaction plot of the estimated marginal means remains essentially identical to that shown in Figure 3.

**Table 2:** Results from the second generalised linear model with the number of observers added to the model.

**Source of variation**	**Wald χ^2^**	**df**	**p-value**
Eyes	0.184	1	0.668
Litter	2.955	1	0.086*
People	0.384	1	0.536
Eyes x litter	1.303	1	0.254
Eyes x people	4.263	1	0.039**

*Marginally non-significant: 0.1>p>0.05; **significant: p<0.05.

## Discussion

In a field experiment on littering behaviour in which we examined the effects of both the presence of signs featuring images of watching eyes and the presence of litter on the ground, we found: (1) that the addition of litter being already present on the ground induced greater littering behaviour; (2) that images of watching eyes reduced littering behaviour, albeit only when there were larger numbers of people in the vicinity; and crucially (3), that there was no evidence that litter on the ground interacted non-additively with images of watching eyes to induce enhanced littering behaviour

.Our first finding, that litter on the ground induces greater littering behaviour compared to the condition in which there was no litter on the ground, replicates the findings from several previous experimental and observational studies [16,19,21,22], and confirms our assumption that we would be able to manipulate littering behaviour by changing the local descriptive norm for littering by altering the cues of previous littering behaviour present in the environment. This demonstration that we could manipulate the local descriptive norm for littering was a critical prerequisite for the success of the study. Although the significant effect of litter became marginally non-significant in the second more complex model including the number of people in the vicinity, we believe that this was due to the reduced power of this latter more complex model to detect an effect of litter. The qualitative pattern was maintained with more people littering when litter was already present on the ground.

Our second finding, that images of watching eyes reduce littering when there are larger numbers of people (6 or more) in the immediate vicinity replicates an eyes effect on littering behaviour in the predicted direction [11], but shows a different interaction with the number of potential real observers around from that seen in previous studies. Ernest-Jones et al. [11] found that posters with images of eyes were only effective at reducing littering on café tables when the café was relatively empty (i.e. below the median number of people present of 46). Other studies have similarly shown that the effect of images of watching eyes is reduced when there are more real observers around [9]. In both cases, the interpretation given for these findings was that in the presence of more real potential observers in the environment the effect of images of eyes became unimportant. Why then did we find that signs with watching eyes were more effective in larger group sizes in the current experiment? It is possibly significant that the eye signs we used were much larger and more obvious in the current experiment than in previous experiments. The sign used in the current study was 90 cm wide x 60 cm tall, whereas the posters used by Ernest-Jones et al. [11] were only 21 cm wide x 29.7 cm tall. It may also be significant that the current study was conducted outside in a public space that participants were passing through as opposed to indoors in a cafeteria or a supermarket queue. When passing through crowded public spaces people tend to avert their gaze from others meaning that although there may be many people present, few may be directly watching each other [23-25]. If few real people are making eye contact this may act to increase the salience of large signs displaying images of watching eyes. Thus, we are suggesting that the relationship between the efficacy of images of watching eyes at modifying behaviour and the number of real people in the vicinity might be non-monotonic. The eye effect might be strongest when people are either alone (the situation in the empty café), or in a large, anonymous crowd (possibly the situation when people were collecting their bikes outside a building in the midst of a large group), and weakest when people are interacting in social groups (possibly the situation when the café was fuller or when people are watching each other in a supermarket checkout queue). Further studies will be needed to explore how the watching eyes effect changes in different group sizes and different social contexts. 

It is noteworthy that the eye image manipulation in the current study made use of pre-exiting signage designed to deter bicycle theft that bore the verbal message, “Cycle thieves we are watching you!” We have previously shown that he same signs produced a significant reduction in thefts of bicycles from their vicinity [12]. However, in that study it was impossible to separate the contributions of the eye images and the verbal message to the observed change in behaviour, and it is possible that the eyes could have simply drawn thieves’ attention to the verbal message rather than altering their behaviour directly. It is therefore interesting that we have found some effects of the same signs on a different anti-social behaviour pattern not alluded to in the signs. This finding supports previous results suggesting that images of eyes can affect behaviour directly, presumably by engaging the psychology of surveillance, rather than by drawing observers’ attention to a verbal message [10,11].

The central aim of the current study was to test the hypothesis that images of watching eyes work by making people behave more normatively as opposed to universally more pro-socially. This hypothesis led to the prediction that when the local descriptive norm was to drop litter, watching eyes would induce increased levels of littering behaviour (Figure 1b). The data provided no support for this prediction. Figure 3 confirms that there is no evidence for the predicted interaction between eyes x litter shown in Figure 1b; if anything, the qualitative trend appears to be in the opposite direction, with the effect of litter on the ground on littering behaviour being attenuated in the presence of eyes rather than exaggerated (Figure 3). Indeed the highest level of littering is shown in the condition with litter on the ground and no eyes present, consistent with the additive model shown in Figure 1a.

The findings of the current study thus concur with those of Powell et al. [9], who observed a positive effect of watching eyes on pro-sociality in a situation (charitable collection buckets in a supermarket) where pro-sociality was not normative. Our current experiment adds to this result by showing that experimentally manipulating what is normative does not seem to moderate the watching eyes effect. This suggests that watching eyes may activate a psychology of reputation whose ultimate origins lie in processes of reputation-based partner choice [13], rather than activating a norm psychology in which punishment for non-normativity is the salient outcome predicted by observation by others (see also Oda et al. [6] for related conclusions). This is not to imply that concerns about normativity are unimportant for human social action - on the contrary, we observed a norm effect in the current study – but rather that the effect of watching eyes is not to heighten normative concerns, but rather to heighten the motivation to behave more pro-socially, independently of local social norms. This finding is potentially important, since it implies that real-world interventions based on ‘watching eyes’ could be effective in settings where pro-sociality is not currently (descriptively) normative, as well as those where it is. 

In conclusion, in a field experiment in which we manipulated cues of locally normative behaviour, we found no evidence to support the hypothesis that images of watching eyes make behaviour more normative. Instead, our data provide tentative support the hypothesis that images of watching eyes induce more pro-social behaviour irrespective of the local descriptive norm. This finding has important implications for the potential use of watching eyes as a cheap intervention to reduce littering since it suggests that the strategy could provide benefits independent of the local littering norm.

## Supporting Information

Data S1 **This file contains information on the contents of the .CSV file supplied in Data S2.**
(DOCX)

Data S2 **A .CSV file containing the data on which the analyses presented in this paper are based.**
(CSV)
