# Supplementary material for: Do Images of ‘Watching Eyes’ Induce Behaviour That Is More Pro-Social or More Normative? A Field Experiment on Littering
Source: PLoS One. 2013 Dec 5;8(12):e82055. doi: 10.1371/journal.pone.0082055 (PMC3855385; doi:10.1371/journal.pone.0082055)
Supplement: Data S1 — This file contains information on the contents of the .CSV file supplied in Data S2. (DOCX) [file pone.0082055.s001.docx]

**Data S1:**

The data on which this manuscript is based are supplied as a .CSV file (Supporting Information: Data S2). The file contains the following 11 columns of data:

1. “Eyes”: 0 = no eyes; 1 = eyes
2. “Litter”: 0 = no litter; 1 = litter
3. “Location”: the name of the location of the observation (6 possible)
4. “Date”: the calendar date of the observation
5. “Time”: the time of the observation (24 hr clock)
6. “Sex”: the apparent sex of the participant (m or f)
7. “AgeRange”: the apparent age range of the participant (categorised as either <18, 18-25, 26-40)
8. “PeopleRange”: the number of people in the vicinity (categorised as either 0, 1-5, 6-10, 11-15 or 16+)
9. “DisposalCode”: 1 = kept on their person; 2 = placed in a nearby litter bin; 3 = left without removing; 4 = thrown on the ground; 5 = placed elsewhere in the vicinity
10. “Littered”: 0 = not thrown on the ground; 1 = thrown on the ground
11. “PeopleBin”: binary categorisation of people in the vicinity (1 = 0-5; 2 = 6+)
